# Shifting landscapes: dynamic changes from pro- to anti-inflammatory leukocyte phenotype in myocardial ischemia/reperfusion injury

**DOI:** 10.3389/fcvm.2025.1596538

**Published:** 2025-06-30

**Authors:** Pia Kröning, Maximilian Mauler, Nancy Schanze, Katharina Naber, Daniela Stallmann, Daniel Duerschmied, Dirk Westermann, Nadine Gauchel

**Affiliations:** ^1^ Department of Cardiology and Angiology, University Heart Center Freiburg-Bad Krozingen, Medical Center – University of Freiburg, Faculty of Medicine, University of Freiburg, Freiburg, Germany; ^2^ Department of Cardiology, Angiology, Haemostaseology and Medical Intensive Care, University Medical Centre Mannheim, Medical Faculty Mannheim, Heidelberg University, Mannheim, Germany; ^3^ European Center for AngioScience (ECAS) and German Center for Cardiovascular Research (DZHK) Partner Site Heidelberg/Mannheim, Mannheim, Germany

**Keywords:** ischemia reperfusion injury, myocardial infarction, neutrophils, monocytes, platelet-leukocyte-complexes, inflammation

## Abstract

**Background:**

The temporal and spatial dynamics of platelet–leukocyte complex (PLC) formation in myocardial ischemia reperfusion injury (I/R injury) are still ill defined.

**Aim:**

To investigate the kinetics and spatial differences of platelet-monocyte (PMC) and platelet-neutrophil (PNC) complex formation over the first 7 days in a mouse model of myocardial I/R injury.

**Methods:**

A time-course study was conducted up to 7 days in order to evaluate immune cell response and cardiac function following myocardial I/R injury in mice. Myocardial I/R injury was induced by ligation of the left anterior descending coronary artery (LAD) for 30 min followed by reperfusion. Using flow cytometry leukocyte and platelet markers were evaluated in the heart, blood, spleen, and bone marrow. Echocardiography was performed in order to measure ejection fraction and fractional shortening which are accepted indicators of cardiac function.

**Results:**

Expression of CD206-Geometric Mean Fluorescence Intensity (GMFI) indicative of an anti-inflammatory phenotype in neutrophils (N2) increased in PNCs at day 7. A statitstically significant decrease in the percentage of anti-inflammatory Ly6C^low^ PMCs was observed as early as day 3, when compared to the baseline value. Flow cytometry analysis showed no significant variations in PNCs or PMCs within the area at risk (AAR) across the specified time points. A rise in neutrophils and monocytes was observed in the AAR, reaching its peak on day 3.

**Conclusion:**

The present study demonstrates that both anti-inflammatory Ly6C^low^ monocytes and N2 neutrophils participate in PLC formation following myocardial infarction (MI) and reperfusion. Our results suggest that the N2 phenotype prerequisite for PLC formation in AAR at day 7. These findings suggest that targeted interventions may be developed to improve outcomes after myocardial I/R injury.

## 1 Introduction

Acute myocardial infarction (MI) is a leading cause of mortality and morbidity worldwide (1). The area at risk (AAR) is the most vulnerable and sensitive region of the myocardium. This is due to its direct dependence on the blood supply from the occluded vessel, resulting in a critical deprivation of oxygen and nutrients. The restoration of blood flow through the culprit coronary artery in a timely manner is the most effective therapy to limit the infarct size and improve the patients outocme. However, sudden reperfusion after a period of tissue ischemia can result in a phenomenon known as ischemia/reperfusion injury (I/R injury). I/R injury has been shown to contribute to as much as 50% of the final infarct size, and is associated with myocardial stunning, arrhythmias and no-reflow (2, 3). The underlying mechanisms are multifaceted and comprise oxidative stress, mitochondrial dysfunction, changes in ion concentrations as well as pH, endothelial activation, and, importantly, a profound immune response (4). Neutrophils and monocytes engage in a dynamic interplay that profoundly influences tissue damage and repair. This process, driven by various signalling cascades, involves interactions between recruited immune cells and the resident endothelium, leading to cellular activation and complex formation. These interactions, in turn, amplify the local immune response and contribute to the progression of inflammation (5–11).

Neutrophils have long been considered as the primary mediators of tissue damage through the release of reactive oxygen species and proteases, but they also exhibit regenerative properties, challenging the conventional paradigm of their role solely in inflammation (6–9). Upon reaching the target tissue, activated neutrophils have been shown to produce high levels of reactive oxygen species (ROS), secrete myeloperoxidase (MPO) and proteases. This process has been demonstrated to exacerbate local vascular and tissue damage (10). This damage promotes the infiltration of activated monocytes, which phagocytose tissue debris and apoptotic neutrophils, leading to the activation of polarized macrophages. This sequence of events establishes the foundation for the essential process of scar formation (11, 12). As with neutrophils, both pro-inflammatory Ly6C^high^ and anti-inflammatory Ly6C^low^ monocyte subsets are present in the blood and the AAR. Pro-inflammatory Ly6C^high^ monocytes have been shown to secrete cytokines that mediate proteolytic degradation of necrotic cardiac tissue, followed by its phagocytosis. Furthermore, a process of differentiation into anti-inflammatory Ly6C^low^ monocytes and Ly6C^low^F4/80^+^ macrophages occurs, which play a critical role in facilitating regeneration and scar formation in the injured myocardium (13, 14).

Platelets, well-known for their role in hemostasis, emerge as pivotal regulators of the immune response post I/R injury (15). Through intricate interactions with neutrophils and monocytes, platelets modulate inflammatory pathways and influence tissue repair processes.

In this context, the detection of platelet-leukocyte complexes (PLCs) represents an interesting observation, although their precise role within the immunobiological processes following I/R injury remains unclear. Recent studies have indicated an elevated prevalence of circulating PLCs in patients with coronary artery disease (16–18). There is evidence that suggests that PLC formation is associated with leukocyte activation, including cytokine release, adhesion molecule and cell surface receptor exposure. Furthermore, this process has been shown to contribute to inflammatory tissue injury (17, 19–21). The targeted reduction of inflammatory cell recruitment and/or PLCs following myocardial infarction (MI) has been shown to be a promising strategy for reducing injury and promoting repair (22, 23). However, such interventions must be meticulously calibrated with regard to their scope and timing, as imbalances in the immune response can result in excessive inflammation, prolonged tissue damage, and adverse left ventricle remodeling (9, 24, 25). Mouse models of cardiac injury, including the ligation of the left anterior descending coronary artery (LAD) serve as preclinical tools to discover new treatment options for post MI healing (26).

While a permanent LAD ligation primarily results in ischemic injury, the transient ligation of the LAD in mice has been shown to resemble contemporary revascularization therapy, thereby encompassing the inflammatory reaction associated with reperfusion injury (26). In order to be of value for preclinical testing, knowledge on the timely regulation of the immune cell landscape including recruitment from hematopoietic organs or storage sites in these MI models is necessary.

The present study investigates the complex dynamic interactions between neutrophils, monocytes, and platelets in the context of I/R injury over the first 7 days, emphasising their dualistic functions in both tissue damage and repair. Cardiac performance was quantified, using ejection fraction (EF) and fractional shortening (FS) on the designated study days. The objective of this study is to elucidate the complex kinetics and interactions of the participating leukocytes underlying the immune response within the first 7 days after I/R injury. This will provide a foundation for the development of novel therapeutic strategies to mitigate I/R injury damage and promote myocardial tissue regeneration.

## 2 Materials and methods

### 2.1 Animals

Nine- to twelve-week-old male C57BL/6N mice were purchased from Charles River (Sulzfeld, Germany). All mice were housed in the local animal facility. All experiments were conducted strictly according to the German animal protection law and in accordance with good animal practice as defined by the Federation of Laboratory Animal Science Associations (https://www.felasa.eu) and the national animal welfare body GV-SOLAS (https://www.gv-solas.de). The examinations undertaken in this study were approved by the federal authorities in Freiburg and the Institutional Review Board (G-17/44).

### 2.2 Murine model of myocardial ischemia reperfusion

All surgeries were performed between 10 a.m. and 2 p.m. to avoid circadian rhythm-associated irregularities according to the guidelines for experimental models of myocardial ischemia and infarction (27). In brief, 9–12 week-old wildtype (WT) mice were anesthetized by intraperitonial (i.p.) injection of 100 mg/kg Ketamine (Ketavet, Pfizer Pharmacia, Berlin, Germany) and 5 mg/kg Xylazine (Rompun, Bayer Vital, Leverkusen, Germany). Analgesia was provided by subcutaneous (s.c.) injection of 0.1 mg/kg buprenorphine. To avoid dehydration 500 µl warm 0.9% NaCl (9 mg/ml) containing 5% glucose (G5%, B. Braun Melsungen, Melsungen, Germany) was injected i.p. Mice were placed on a heating pad to maintain body temperature at 37°C. The operation table was tilted 20°C to allow visualization of the vocal cords and mice were intubated under sight. Oxygen saturation, heart rate, and respiratory rate were monitored throughout the procedure by a MouseOX system (Starr Life Sciences, Oakmont, PA). Anesthesia was maintained by addition of 1.2% isoflurane (Abbott, Wiesbaden, Germany) during surgery. After right lateral positioning of the animal, left lateral thoracotomy was performed and the LAD was identified. To induce ischemia an 8.0 nylon suture (Prolene, Ethicon, Norderstedt, Germany) was placed around the vessel and a loose loop was formed. A fine bore 0.61 mm polythene tube was placed on the LAD and the loop was tied. Ligation was visually confirmed by paling of the left ventricular anterior wall. The tube was removed after 30 min to allow reperfusion of myocardium. After evacuation of the pneumothorax, chest and skin wounds were closed using a 6–0 prolene suture (Prolene, Ethicon, Norderstedt, Germany).

Mice were monitored until spontaneous breathing occurred, extubated and placed under a heating lamp. Analgesia with Buprenorphine was administered every 6 h.

### 2.3 Flow cytometry

#### 2.3.1 Murine blood samples

Blood from WT mice was obtained by cardiac puncture using a 30—gauge needle coated with unfractionated heparin (B. Braun Melsungen, Melsungen, Germany). 1 ml blood was immediately transferred to 100 µl enoxaparine 1.5 μg/μl (Sanofi Aventis, Frankfurt a. M., Germany). 100 µl of this solution were diluted with 500 µl phosphate buffered saline containing 0.9 mmol/L calcium, 0.5 mmol/L magnesium (PBS+/+), (PBS, Lonza, Verviers, Belgium), and 0.1% bovine serum albumin (BSA, BSA, SERVA Electrophoresis, Heidelberg, Germany). 5 µl of this dilution were incubated with 95 µl red-blood-cell- lysis buffer (BD Bioscience, Heidelberg, Germany) for 5 min and leukocytes were counted in a Neubauer chamber. 90 µl diluted blood was mixed with 10 µl antibody mix and incubated for 15 min in the dark at room temperature. Stained whole blood samples were mixed gently with 400 µl 37°C warm 1× Phosflow Lyse/Fix buffer (BD Bioscience, Heidelberg, Germany) and incubated for 20 min at room temperature. Gating strategy Supplementary Figure S2.

To evaluate leukocyte subsets, platelet neutrophil complexes and platelet monocyte complexes after I/R injury, 10 µl of the following 1:10 antibody—PBS+/+—BSA dilution was used: anti-CD45.2AmCyan (clone 104), anti-CD206 PacBlue (clone 19.2), anti-CD11b-APCCy7 (clone M1/70), anti-CD115-APC (clone CSF-1R), anti-CD3-FITC (clone 145–2C11), anti-CD19 PE/Cy7 (clone 6D5), 4/80 PE (clone BM8.1), anti-CD41aFITC (clone MWReg30), anti-Ly6G PECy7 (clone RB6-8C5), anti-CD45.2AmCyan (clone 104), anti-CD11a/CD18-PerCPCy5.5 (clone H155–78; all BioLegend, Fell, Germany), anti-CD162-PacificBlue (clone 2PH1), CD206 PerCPCy5.5 (clone C068C2) and CD11bAPC (clone M1/70). Gating strategy Supplementary Figure S3.

#### 2.3.2 Tissue analyses

##### 2.3.2.1 Heart

**Figure 1:**
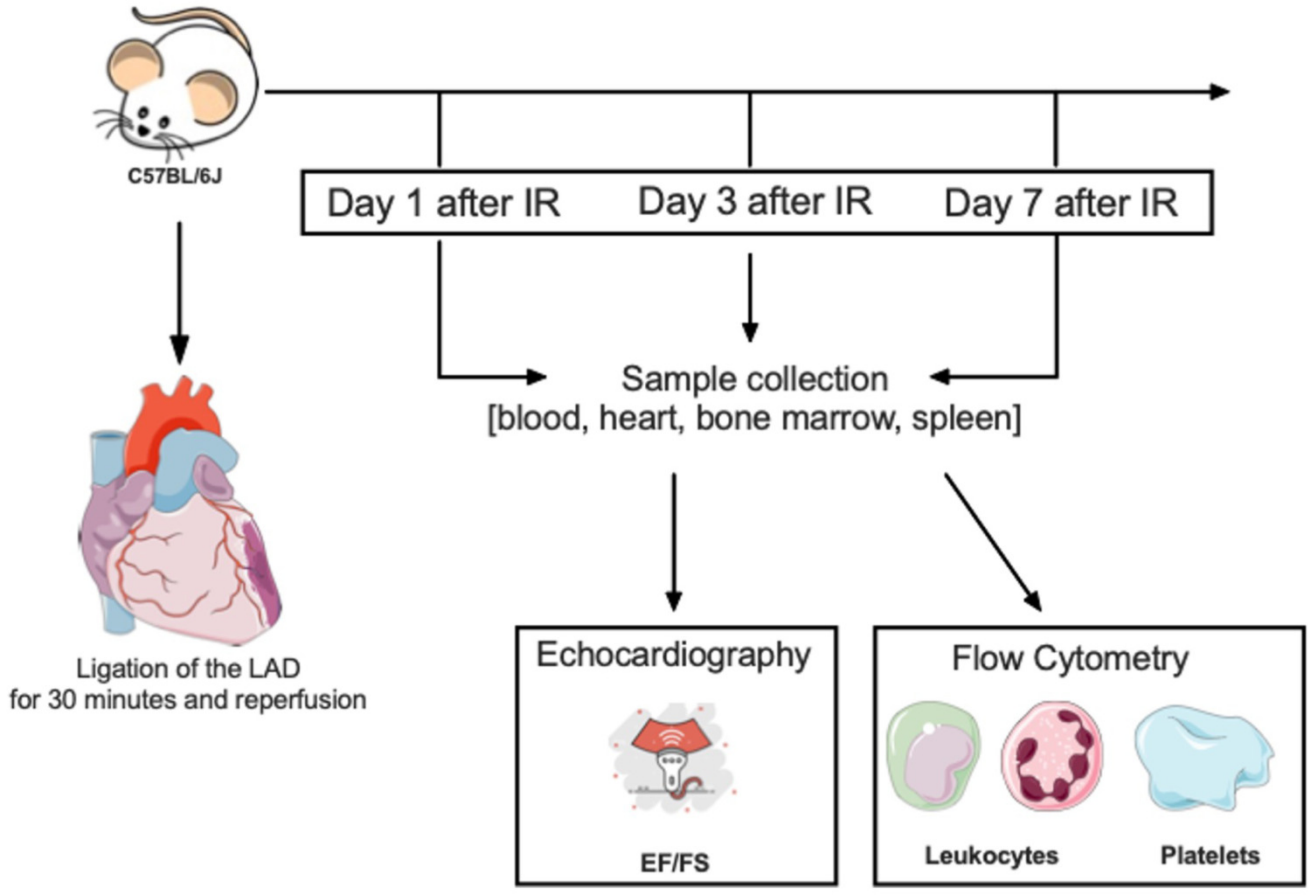
Model structure—schematic time course. In our experimental setup we used 9–12 weeks old C57BL/6J wild-type mice. At day of surgery the LAD was ligated for 30 min with subsequent reperfusion. There was a division into three different subgroups, named according to the respective observation periods, 24 h, 3 and 7 days after I/R injury. A sample collection was performed, including blood, heart tissue, bone marrow and the spleen. Flow cytometry was used to analyze different types of neutrophils, monocytes, macrophages and thrombocytes. In addition, we performed echocardiography to measure cardiac function by means of ejection fraction and fractional shortening.

After 1, 3 or 7 days of reperfusion (Figure 1) hearts were perfused with 0.9% NaCl to remove remaining blood, excised and put into cold PBS+/+. The heart was then divided into posterior and anterior walls. The area at risk (AAR) was defined as the anterior wall apical to the placed ligature, in the region of the LAD supply, and transferred into 1 ml PBS+/+ containing 125 U collagenase XI, 30 U DNase I, 30 U hyaluronidase (all Sigma-Aldrich CHEMIE, Steinheim, Germany), 5 mmol/L CaCl2, 450 U collagenase I (both Biochrom, Berlin, Germany) and 20 mmol/L HEPES (Carl Roth, Karlsruhe, Germany) and homogenised with a scalpel. The homogenate was incubated on a shaker for 1 h (37°C, 700 rpm) and subsequently passed through a 40 μm cell strainer (Becton Dickinson, Heidelberg, Germany) into phosphate buffered saline without calcium and magnesium (PBS−/−) containing 0.1% BSA (FACS Buffer). After centrifugation (4°C, 500*g*, 7 min), the cells were washed and centrifuged again. Unspecific Fc Receptor—mediated antibody binding was blocked by incubation with an anti—mouse CD16/CD32 antibody (Fc Receptor block, eBioscience, Frankfurt, Germany). After addition of 200 μl FACS buffer, 90 μl of the cell suspension was incubated with flow cytometry antibodies (anti-Ly6C-FITC (clone AL-21), anti-MHCII-PerCPCy5.5 (clone M5114.15.2; Life Technologies, Darmstadt, Germany), anti-CD41aPE (clone MWReg30), anti-CD206 PacBlue (clone 19.2), anti-CD11b-APCCy7 (clone M1/70), anti-F4/80-PECy7 (clone BM8), anti-CD45.2AmCyan (clone 104), anti-CD19-PE (clone eBio1D3), anti-CD3e-PE (clone 145-2C11), anti-B220-PE (clone RA3-6B2), anti-CD90.2-PE (clone 53-2.1), anti- CD49b-PE (cloneDX5), anti-NK1.1-PE (clone PK136) and anti-Ly6G-PE (clone 1A8; all Becton Dickinson) or 30 min on ice in the dark. Samples were washed twice, resuspended in 300 μl FACS buffer and stored in the dark until FC analysis. Gating strategy Supplementary Figure S4.

##### 2.3.2.2 Spleen

The spleen was excised 1, 3 and 7 days post I/R injury and half of the spleen was passed through a 40 µm cell strainer. The resulting cell suspension underwent centrifugation (4°C, 5 min, 1,300*g*), red blood cell lysis, and further washing steps. After resuspension in FACS buffer (PBS−/−; 0.1% BSA), the suspension was filtered again, stained with a 50 µl antibody mix for 25 min on ice in the dark, washed twice, and resuspended in 300 µl FACS buffer for analysis. Gating strategy Supplementary Figure S2.

##### 2.3.2.3 Bone marrow

The femur was harvested 1, 3, or 7 days after I/R injury, rinsed with PBS−/−, and the bone marrow was flushed and filtered through a 40 µm cell strainer. The strainer was rinsed with 25 ml PBS−/−, and the resulting cell suspension was centrifuged (4°C, 5 min, 1,300*g*), and the supernatant discarded. Cells were stained with a 50 µl antibody mix for 25 min on ice in the dark, washed twice with 1 ml FACS buffer, recentrifuged (4°C, 500*g*, 3 min), and resuspended in 300 µl FACS buffer for further analysis. Gating strategy Supplementary Figure S2.

Antibody mix bone marrow and spleen contained the following monoclonal anti—mouse antibodies 1:100 diluted in FACS buffer: anti-CD45.2AmCyan (clone 104), anti-CD206 PacBlue (clone 19.2), anti-CD11b-APCCy7 (clone M1/70), anti-CD115-APC (clone CSF-1R), anti-CD3-FITC (clone 145–2C11), anti-CD19 PE/Cy7 (clone 6D5), 4/80 PE (clone BM8.1), anti-CD41aFITC (clone MWReg30), anti-Ly6G PECy7 (clone RB6-8C5), anti-CD45.2AmCyan (clone 104), anti-CD11a/CD18-PerCPCy5.5 (clone H155–78; all BioLegend, Fell, Germany), anti-CD162-PacificBlue (clone 2PH1), CD206 PerCPCy5.5 (clone C068C2) and CD11bAPC (clone M1/70).

Data were acquired on a BD FACSCanto II (BD Bioscience, Heidelberg, Germany) and analyzed with FlowJo v10 software (Tree Star, Ashland, OR USA).

### 2.4 Echocardiography

Echocardiography was performed either on day 1, 3 or 7 as previously described (28). In brief, mice were anesthetized by 2% isoflurane i.n. and placed on a heating pad. Echocardiography loops were recorded in B and M modes in longitudinal and short axis views on a Vevo 3100 equipped with a MX550D transducer (both FUJIFILM VisualSonics, Inc., Toronto, ON, Canada). Heart rate was monitored during the procedure. Systole and diastole were defined based on concomitant electrocardiography (ECG) recordings. The end-systolic time point for left ventricular (LV) diameter measurement was defined as the maximum of ventricle contraction just before complete closure of the aortic valve. End-diastole was defined as the maximum of LV dilation and filling just before mitral valve closing (when visible) and aortic valve opening. Left ventricular ejection fraction (LVEF) was determined by LV tracing relating end systolic LV area as the minimal LV cross-sectional area to end-diastolic LV area as the maximum LV cross-sectional area in long axis views. FS was assessed from short axis M-mode using VevoLab 5.5.1 software (FUJIFILM VisualSonics).

### 2.5 Statistical analysis

Statistical analyses were performed using GraphPad Prism version 8. The Shapiro–Wilk test was employed to assess data for normality.

Normally distributed data are presented as mean ± standard error of the mean (SEM), while non-normally distributed data are reported as median with interquartile range (IQR). Statistical outliers were identified using the ROUT test. Comparisons of normally distributed data were conducted using one-way ANOVA followed by Tukey's multiple comparisons test. Non-normally distributed data were analyzed using the Kruskal–Wallis test.

## 3 Results

### 3.1 Neutrophils and monocytes increase after I/R injury

**Figure 2:**
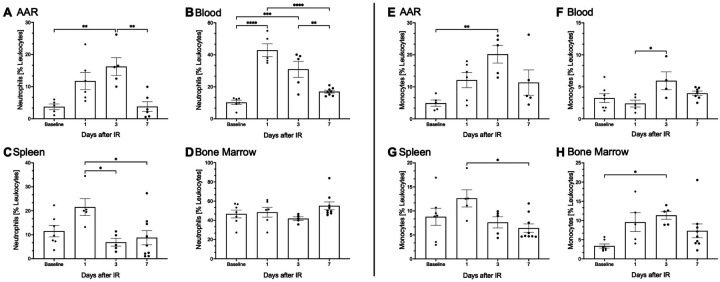
I/R injury innitiates an immediate primary immune response. Surgical ligation of the left anterior descending artery (LAD) was performed in 9- to 12-weeks-old male C57BL/6N mice for 30 min, followed by reperfusion. **(A–H)** Kinetic analysis of neutrophils and monocytes depicted as percentage of the entire leukocyte population across blood, heart, spleen, and bone marrow at baseline and 1-, 3-, and 7-days post (I/R) injury, using flow cytometry. Pre-sorting of leukocyte population by using forward scatter (FSC) and side scatter (SSC). Neutrophils and monocytes are represented on the *Y*-axis, with time points shown on the *X*-axis. **(A–D)** illustrate the kinetics of CD45+, CD3−, CD19−, CD11b+, CD115+, Ly6C intermediate neutrophils in **(B)** blood and lineage+, CD11b+, Ly6C intermediate neutrophils in **(A)** heart, **(C)** spleen and **(D)** bone marrow. **(E–H)** Shows the analysis of CD45+, CD3−, CD19−, CD11b+, CD115+, Ly6C high/low monocytes in **(F)** blood, lineage+, CD11b+, F4/80−, CD115+, Ly6C^high/low^ monocytes in **(E)** heart, **(G)** spleen, and **(H)** bone marrow at baseline and 1-, 3-, and 7-days post- (I/R) injury. Dots are individual values (one mouse), average *n* = 6 per group. Data are shown in mean with standard error of the mean (SEM). *P*-values are based on ordinary two-way ANOVA Tukey's multiple comparison test. Bracket accompanied with asterisks indicate statistical significance: *p ≤ 0.05, **p ≤ 0.01, ***p ≤ 0.001, ****p ≤ 0.0001.

A significant increase in the percentage of neutrophils of the total leukocytes was observed 1 day after I/R injury in both, blood (9.1% ± 1.5 vs. 42.8% ± 3.6, *p* ≤ 0.001; Figure 2B) and spleen (11.5 ± 2.3 vs. 21.5% ± 3.1; Figure 2C). Additionally, a significant rise of the proportion of neutrophils was detected in the AAR 3 days post I/R injury (3.7% ± 0.8 vs. 16.2% ± 2.4, *p* ≤ 0.01; Figure 2A). By 7 days after I/R injury, the percentage of neutrophils in the AAR, blood, and spleen returned to the baseline range (10%–15%) (6). In contrast, neutrophil proportions in the bone marrow showed only minor changes over the observation period. Monocyte proportion increase appeared in parallel to that of neutrophils. Three days post I/R injury, a significant increase in the monocyte percentage was observed in the AAR (4.9% ± 0.9 vs. 20.1% ± 2.4, *p* ≤ 0.01; Figure 2E), blood (2.3% ± 0.5 vs.7.5% ± 1.3, *p* ≤ 0.05; Figure 2F) and bone marrow (3.3% ± 0.5 vs. 11.3% ± 1.8, *p* ≤ 0.05; Figure 2H). In the spleen 1 day after I/R injury a significant increase of monocyte proportion was detected 1 day after I/R injury (12.61 ± 1.6 vs. 6.4% ± 0.8, *p* ≤ 0.05; Figure 2G). Similarly, by 7 days post I/R injury, monocyte proportions in all examined tissues had nearly returned to baseline levels.

### 3.2 Functional subtypes of monocytes influence inflammation and regeneration of the AAR

**Figure 3:**
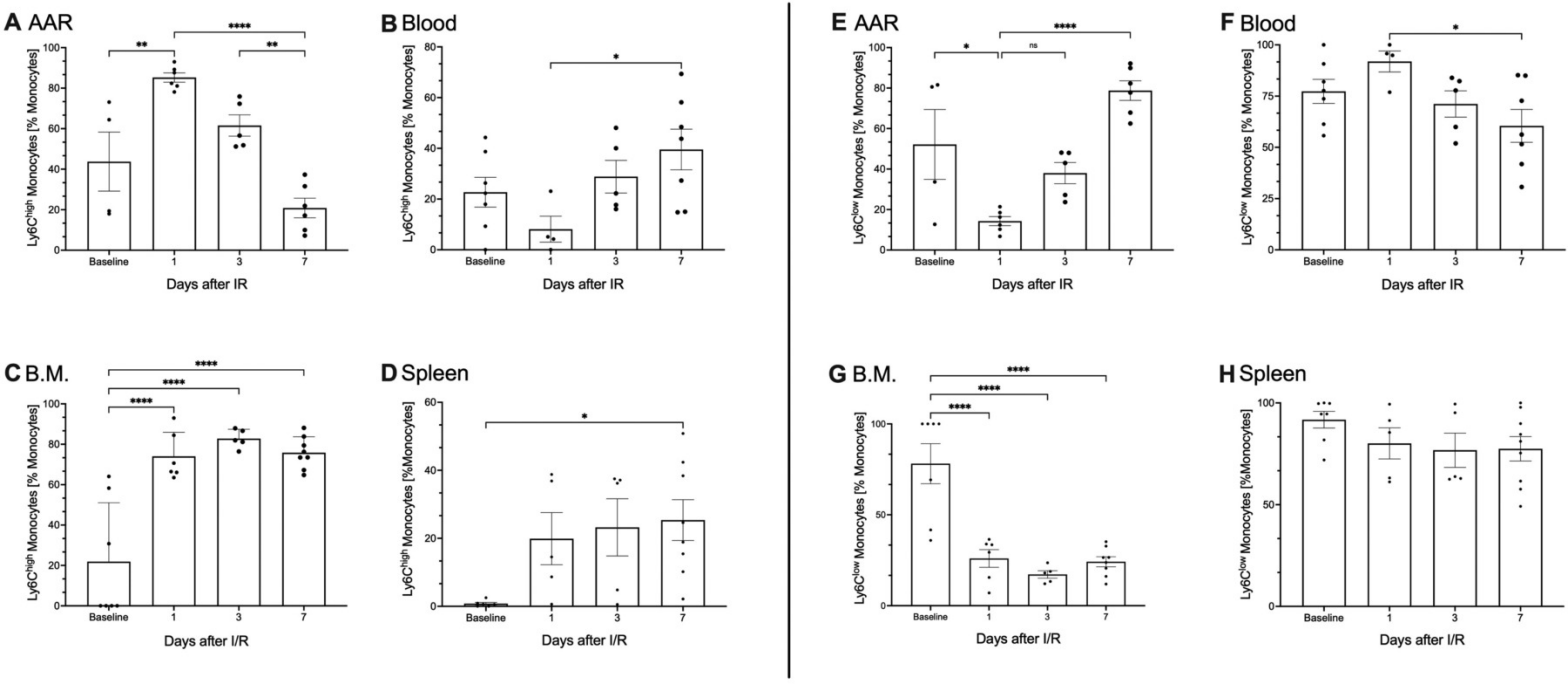
Functional subtypes of monocytes influence inflammation and regeneration of the AAR. As part of the primary immune response following (I/R) injury, both pro-inflammatory (Ly6C^high^) and anti-inflammatory (Ly6C^low^) monocytes are activated in the analyzed tissues, including blood, heart, spleen, and bone marrow. **(A–H)** Kinetic analysis of pro-inflammatory (Ly6C^high^) and anti-inflammatory (Ly6C^low^) monocytes as percentage of the total measured monocyte population across blood, heart, spleen, and bone marrow at baseline and 1-, 3-, and 7-days post (I/R) injury, using flow cytometry. Pre-sorting of leukocyte population by using forward scatter (FSC) and side scatter (SSC). Proinflammatory CD45+, CD3−, CD19−, CD11b+, CD115+, Ly6C^high^ monocytes in **(B)** blood and lineage-, CD11b+, F4/80−, CD115+, Ly6C^high^ monocytes in **(A)** heart, **(C)** spleen, and **(D)** bone marrow, antiinflammatory CD45+, CD3−, CD19−, CD11b+, CD115+, Ly6C^low^ monocytes in **(F)** blood, lineage+, CD11b+, F4/80−, CD115+, Ly6C^low^ monocytes in **(E)** heart, **(G)** spleen, and **(H)** bone marrow are represented on the *Y*-axis, with time points shown on the *X*-axis Dots are individual values (one mouse), average *n* = 6 per group. Data are shown in mean with standard error of the mean (SEM). *P*-values are based on ordinary two-way ANOVA Tukey's multiple comparison test. Bracket accompanied with asterisks indicate statistical significance: *p ≤ 0.05, **p ≤ 0.01, ****p ≤ 0.0001.

Phenotypically and functionally distinct monocyte subtypes were differentiated using Ly6C expression. One day post I/R injury, the percentage of pro-inflammatory Ly6C^high^ monocytes peaked significantly in the AAR (43.7% ± 12.6 vs. 85.2% ± 2.1, *p* ≤ 0.01; Figure 3A). In contrast the percentage of anti-inflammatory Ly6C^low^ monocytes showed a marked decrease at this time, followed by a significant increase in the AAR 7 days after I/R injury (14.2% ± 2.03 vs. 78.8% ± 4.4, *p* ≤ 0.001; Figure 3E). Concurrently a percentage decline in pro-inflammatory Ly6C^high^ monocytes was observed in the blood, followed by a significant rise 7 days post I/R injury (8.11% ± 4.4 vs. 39.5% ± 7.4, *p* ≤ 0.05; Figure 3B). Conversely, the percentage of anti-inflammatory Ly6C^low^ monocytes in the blood increased significantly 1 day after I/R injury before returning to baseline values (96.9% ± 1.3 vs. 60.4% ± 7,4; *p* ≤ 0.05; Figure 3F). Pro-inflammatory Ly6C^high^ monocytes also showed a significant percentage increase in the spleen (1.3% ± 0.4 vs. 22.6% ± 5.6, *p* ≤ 0.05; Figure 3D) and bone marrow (21.8% ± 10 vs. 72.8% ± 6.8; Figure 3D) within the first 7 days post I/R injury. Additionally, the bone marrow exhibited consistently lower proportions of anti-inflammatory Ly6C^low^ monocytes within the first 7 days after I/R injury compared to the baseline (78.1% ± 10.2 vs. 21.2% ± 4,68, *p* ≤ 0.0001; Figure 3G).

### 3.3 Sterile inflammation leads to complex formation between activated leukocytes and platelets

**Figure 4:**
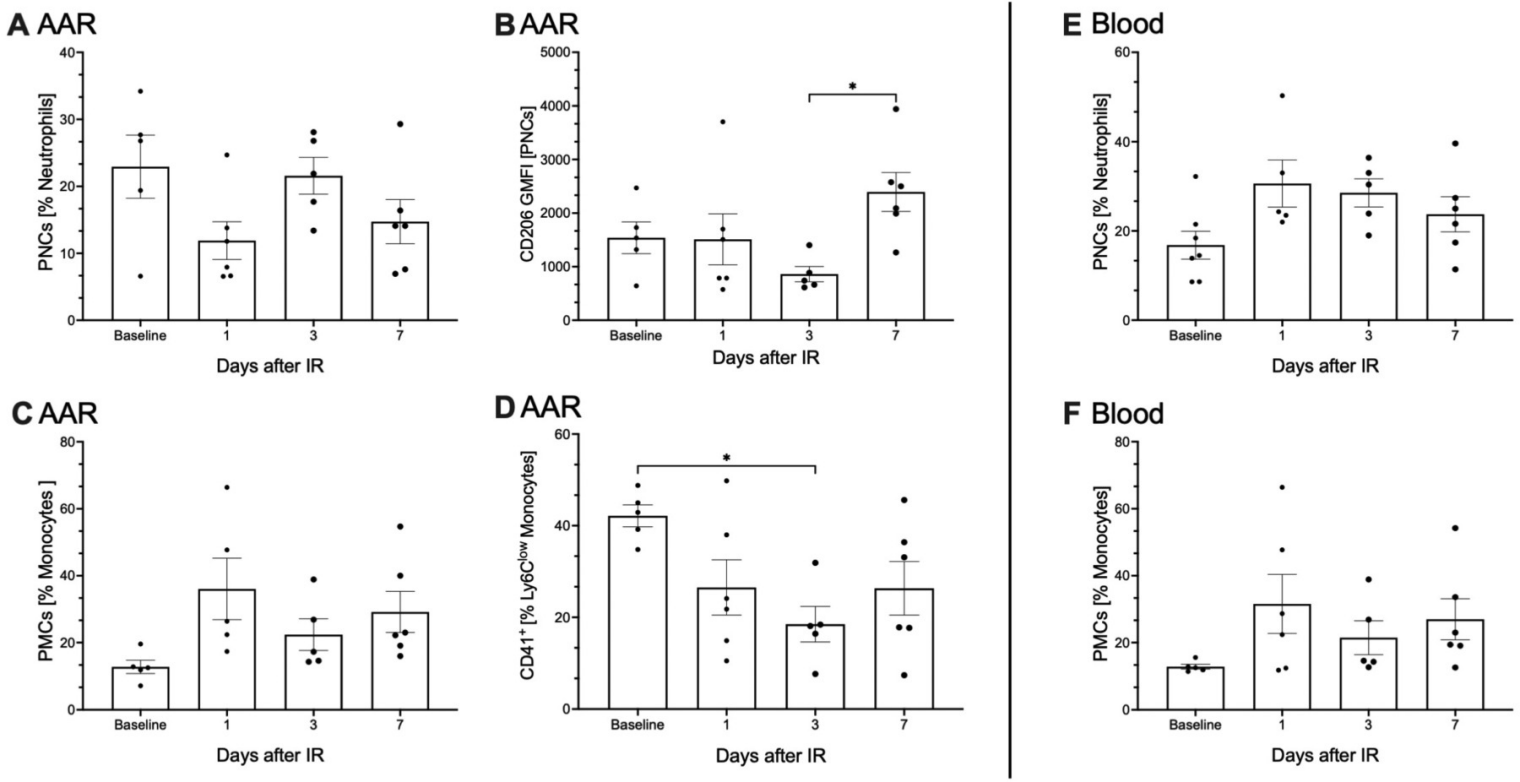
Sterile inflammation leads to complex formation between activated leukocytes and platelets. Due to sterile inflammation following (I/R) injury, activated platelets are recruited to facilitate migration into the area at risk (AAR) **(A–D)**, by forming complexes with neutrophils and monocytes. These complexes were also detected in the blood **(E,F)**. The analysis of these complexes was performed using flow cytometry. Pre-sorting of leukocyte population by using forward scatter (FSC) and side scatter (SSC) was performed. **(A)** depicts lineage+, CD11b+, Ly6C intermediate, CD41 + platelet-neutrophil complexes in the AAR, while **(E)** shows Ly6G+, CD41 + platelet-neutrophil complexes in the blood, expressed as a percentage of neutrophils on the *Y*-axis, with time points displayed on the *X*-axis. No significant differences were observed between time points. To further specify neutrophil subpopulations, the neutrophil-specific antibody CD206, using Geometric mean of fluorescence intensities (GMFI) was employed. In the AAR **(B)**, an anti-inflammatory subpopulation of platelet-neutrophil complexes (lineage+, CD11b+, Ly6C intermediate, CD41+, CD206+) was identified. This subpopulation demonstrated a significant increase 7 days post-I/R, *p*-value ≤ 0.05. In the AAR **(C,D)**, lineage+, CD11b+, F4/80−, CD115+, Ly6C^high/low^, CD41+, platelet-monocyte complexes were identified and expressed as a percentage of monocytes on the *Y*-axis, with time points displayed on the *X*-axis. In the AAR **(D)**, a significant decline *p*-value ≤ 0.05, in the anti-inflammatory lineage+, CD11b+, F4/80−, CD115+, Ly6C^low^ platelet-monocyte complexes was observed compared to baseline. No measurable changes in platelet-monocyte complexes were detected in the blood **(F)**. **(A–F)** Dots are individual values (one mouse), average *n* = 6 per group. Data are shown in mean with standard error of the mean (SEM). *P*-values are based on ordinary two-way ANOVA Tukey's multiple comparison test. Bracket accompanied with asterisks indicate statistical significance: *p ≤ 0.05.

In the blood, there was a marked increase observed in the proportion of platelet-neutrophil-complexes (PNCs) of the total neutrophils following I/R injury (16.8% ± 2.9 vs. 30.6% ± 4.7; Figure 4E). Additionally, changes were observed in the AAR (11.9% ± 2.6 vs. 21.6% ± 2.5; Figure 4A). However, when examining pro- and anti- inflammatory PNC subtypes, identified by neutrophil surface antigen CD206, GMFI, defined CD206^−^ as proinflammatory (N1) and CD206^+^ as anti-inflammatory (N2) neutrophils. At day 7 after I/R injury a significant increase in CD206-GMFI was detected on N2 PNCs in the AAR (GMFI 861 ± 128 vs. 2,395 ± 332, *p* ≤ 0.05; Figure 4B). Similarly, platelet–monocyte-complexes (PMCs) were identified in both, the AAR and blood (Figures 4C,F). Here also anti -inflammatory Ly6C^low^ PMCs were distinguished.

In the AAR, a statistically significant reduction in the proportion of anti-inflammatory Ly6C^low^. PMCs was observed on day 3 post I/R injury (42.1% ± 2.2 vs. 18.5% ± 3.5, *p* ≤ 0.05; Figure 4D). In blood, clear changes of PMC proportions were also observed, although not statistically significant (18.8% ± 5.7 vs. 36.1% ± 8.2; Figure 4F).

### 3.4 Changes in EF und FS following I/R injury

**Figure 5:**
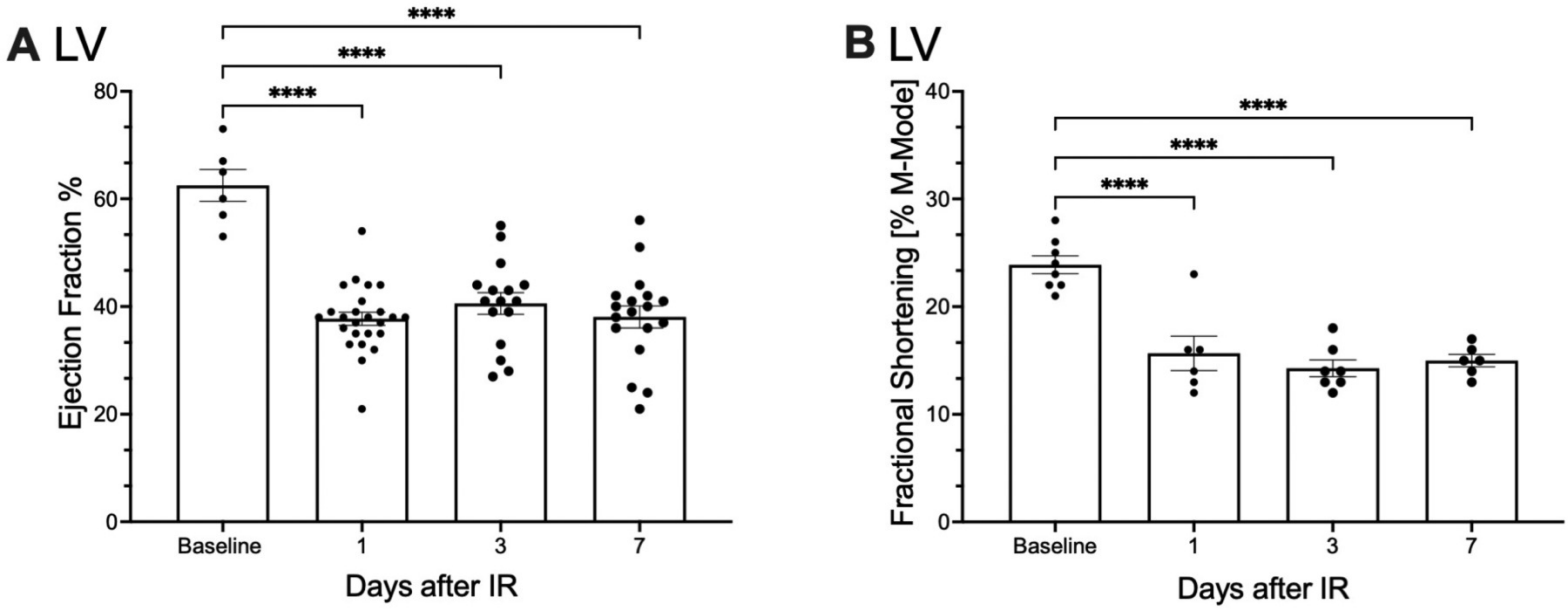
Myocardial function is impaired after I/R injury. Echocardiographic assessments were performed on operated C57BL/6N mice at 1, 3, and 7 days post- (I/R) to evaluate cardiac function and determine the impact of the induced ischemic area. **(A)** Depicts the ejection fraction (EF) as a percentage on the *Y*-axis, with the time points of measurement displayed on the *X*-axis. **(B)** Illustrates the fractional shortening (FS) as a percentage on the *Y*-axis and its temporal progression on the *X*-axis. **(A, B)** Dots are individual values (one mouse), **(A)** average *n* = 14, **(B)** average *n* = 7. Data are shown in mean with standard error of the mean (SEM). *P*-values are based on ordinary two-way ANOVA Tukey's multiple comparison test. Bracket accompanied with asterisks indicate statistical significance: ***p ≤ 0.001.

To confirm the success of the surgery to induce myocardial I/R injury, echocardiographic measurements, including EF and FS, were conducted with the studied animals. A significant reduction in the EF was observed 1 day post I/R injury as compared to baseline, with the decline persisting over the whole observation period of 7 days (62.5% ± 2.7 vs. 37.2% ± 1.2–38.1% ± 2.0, *p* ≤ 0.0001; Figure 5A). Similarly, FS was significantly reduced in mice following I/R injury compared to baseline levels and remained diminished throughout 7 days post I/R injury (23.9% ± 0.8 vs.15.7% ± 1.5%–15.0% ± 0.5, *p* ≤ 0.0001; Figure 5B).

## 4 Discussion

### 4.1 I/R injury leads to an immediate systemic activation of the primary immune response

Within the first 3 days after I/R injury, there is a continuous increase in neutrophils in the AAR, reaching a significant maximum on the third day, Figure 2A. Neutrophils, stimulated by pro-inflammatory cytokines migrate into the AAR via diapedesis and undergo phagocytosis before apoptosis (7, 29). While neutrophil recruitment typically peaks within 24 h of inflammation, I/R injury demonstrates distinct dynamics with an early increase in neutrophil proportions at day 1 post I/R injury and sustained elevations throughout 3 days (29, 30). The persistence of neutrophils aligns with rising leukocytes in AAR and is likely driven by excessive cytokine release, characteristic for reperfusion injury, establishing a feedback loop that amplifies neutrophil recruitment to the AAR (3, 31, 32). Another possible reason for the persistent increase in neutrophils is their prolonged lifespan. Previous data suggested that neutrophils in mice and humans have a lifespan of just 1.5–8 h (5, 33). However, more modern methods have made it possible to measure neutrophils *in vivo*. A study measured the lifespan of neutrophil granulocytes as up to 12.5 h in mice and up to 5.4 days in humans (33, 34). It has also been demonstrated that activated and primed neutrophils that reach the site of inflammation have a longer lifespan (35, 36). Furthermore, it has been demonstrated, that fully mature circulating neutrophils can proliferate further. These cells also have the ability to proliferate, prime and persist in inflamed tissue (10, 35, 36). Once in the tissue, activated neutrophils generate high levels of reactive oxygen species, secrete myeloperoxidase (MPO) and proteases, thereby exacerbating local vascular and tissue damage (10). The result is the infiltration of monocytes, which phagocytose tissue debris. Apoptotic neutrophils, thereby activating polarised macrophages (12). These processes form the basis of the necessary scar formation. Neutrophils play a key role in cardiac healing, but an unbalanced and exaggerated immune response after I/R exacerbates tissue damage. Unbalanced and exaggerated immune response after I/R exacerbates tissue damage, leading to maladaptive remodeling (11, 13). Our data also show a significant increase in the proportion of neutrophils in the spleen 1 day after I/R injury, suggesting its involvement in neutrophil recruitment Figure 2C (37, 38). In contrast, the bone marrow displayed a uniform distribution of neutrophils over the duration of our experiment, indicating a balanced turnover of cell degradation, recruitment, and production Figure 2D. These findings suggest that both the bone marrow and spleen act as key reservoirs for neutrophils. The bone marrow continuously produces neutrophils from myeloid progenitor cells, a process regulated by factors such as Granulocyte-Colony Stimulating Factor (G-CSF), Interleukin 17A (IL-17A), and Interleukin 23 (IL-23) (39). G-CSF is the main regulator of granulopoiesis, encouraging the growth, development, and movement of neutrophils from myeloid progenitor cells in the bone marrow (40). IL-17A enhances the production of G-CSF and chemokines by stimulating stromal and myeloid cells, thereby indirectly boosting neutrophil production and recruitment. IL-23 has an indirect effect on granulopoiesis by promoting the expansion of Th17 cells, which activate the IL-17A/G-CSF axis (41). Furthermore, neutrophils themselves contribute to hematopoietic production by releasing Tumor Necrosis Factor-alpha (TNF-α), which stimulates vascular and hematopoietic regeneration in the bone marrow (40). Neutrophils also undergo breakdown in the bone marrow (41). Monocytes are also recruited to the AAR, peaking alongside neutrophils Figures 2E–H. These monocytes, likely originating from the bone marrow and spleen, play a key role in phagocytosing tissue debris and apoptotic neutrophils. These processes also activate polarized macrophages (12). The kinetics of monocyte recruitment follow a similar pattern to the observed neutrophils. Monocytes also show a significant increase in bone marrow, observed as early as 24 h post I/R injury, peaking at day 3, Figure 2H. This suggests that monocytes are newly formed and released from the bone marrow in response to I/R injury.

The spleen contributes to the early monocyte recruitment within 24 h post I/R injury, Figure 2G. It is estimated, that the spleen contributes approximately 50% of the monocytes, that migrate into the infarcted myocardium (42).

Together, these findings underscore the pivotal roles of neutrophils and monocytes in I/R injury, where a dysregulated immune response exacerbates tissue damage and contributes to maladaptive cardiac remodeling. The bone marrow and spleen play essential roles in the mobilization and clearance of activated leukocytes, as evidenced by our observations across the analyzed time points. Notably, neutrophils emerge as integral players in cardiac repair; however, their disproportionate and overactive immune response following I/R injury amplifies tissue damage and drives maladaptive remodeling (11, 13).

### 4.2 Dynamic interplay of neutrophil and monocyte subtypes: key players in I/R injury and cardiac repair

Upon closer examination N1 and N2 neutrophils were differentiated based on their surface expression of the antigen CD206. CD206 expression on neutrophils in the AAR tended to increase on day 7 post I/R injury, Supplementary Figure S1, indicating a phenotypic change of the present neutrophils into an N2 neutrophil subtype. This functional and phenotypic plasticity, as observed in our data, aligns with earlier descriptions of neutrophil alterations (43). It suggests, that neutrophils are not just important during inflammation, cytokine release, cell death and phagocytosis, but also play an important role in regeneration of the damaged tissue. We assume that activated neutrophils exhibit a longer lifespan (33, 35), Figure 2A, and therefore are capable of undergoing a phenotypic change, redefining their relevance in inflammation and repairment after I/R injury. Furthermore, depletion of neutrophils does not appear to be a rational approach to prevent excessive inflammation. Recent echocardiographic data from neutrophil—depleted mice show impaired myocardial repair, with reduced contractility and increased collagen deposition, leading to fibrosis (9). These findings highlight the therapeutic potential of targeting the inflammatory phase and modulating N1 neutrophils to mitigate reperfusion injury, supported by neuroprotective effects of N2 neutrophils in neurological studies (44). Like neutrophils, monocyte subtypes were differentiated based on their inflammatory profiles. pro-inflammatory Ly6C^high^ monocytes showed a significant increase in the AAR within 24 h post I/R injury, Figure 3A. These cells produce cytokines, facilitate proteolytic degradation of necrotic material, and phagocytose cellular debris (14). Over time, pro-inflammatory Ly6C^high^ monocytes transition into anti-inflammatory Ly6C^low^ monocytes, promoting the differentiation of regenerative Ly6C^low^ F4/80^+^ macrophages essential for tissue repair (45). In the AAR, anti-inflammatory Ly6C^low^ monocyte proportion significantly increased by day 3 after I/R injury, peaking at day 7, Figure 3E. This transition mostly reflects the differentiation of resident pro-inflammatory Ly6C^high^ monocytes, whereas the recruitment through blood is playing a minor role, as supported by kinetic data, Figure 3F. Unlike findings in the spleen, Figures 2D,H, no significant differentiation was observed in splenic pro-inflammatory Ly6C^high^ and anti-inflammatory Ly6C^low^ monocyte subtypes. However, bone marrow analysis revealed distinct kinetics, with pro-inflammatory Ly6C^high^ and anti-inflammatory Ly6C^low^ monocytes stabilizing within 7 days post I/R injury, Figures 3C,G. This suggests that monocyte subtypes recruited to the AAR partly originate from the bone marrow (45, 46).

The data indicate that neutrophils remain in the AAR longer than previously assumed. Importantly, anti-inflammatory and regenerative functions are evident not only in monocytes and macrophages but also in neutrophils. This suggests that the extent of reperfusion injury is influenced more by the phenotypic characteristics of recruited cells than by their overall numbers. Our findings further validate the dynamic kinetics and phenotypic transitions of monocytes in the AAR described in the literature (46, 47).

The shift from pro-inflammatory Ly6C^high^ to anti-inflammatory Ly6C^low^ monocytes three after post I/R injury, along with normalization by day 7, is consistent with established models. Moreover, our results highlight the role of the bone marrow in supporting these transitions and delivering functionally altered monocytes to the affected tissue.

This underscores the importance of understanding immune cell subtypes in developing targeted therapies for I/R injury.

### 4.3 Platelet-leukocyte interactions in I/R injury: insights into complex formation and functional implications

Our data confirm the presence and kinetic change of PNCs and PMCs in the AAR (Figure 4). While these complexes do not show significant changes at first glance, further analysis reveals important trends in functional phenotypes. Notably, the surface expression of the neutrophil—specific antigen CD206 increases significantly in PNCs 7 days post I/R injury (Figure 4B), suggesting an elevated presence of N2 neutrophils, some bound to platelets. This trend indicates that N2 PNCs might increase further at later time points, such as 10–21 days post I/R injury. Despite this potential, current evidence suggests that PNCs themselves have no direct functional impact on ischemic myocardium after I/R (48). Following I/R injury, platelets are activated alongside leukocytes (49, 50). Activated neutrophils express the P-selectin glycoprotein ligand-1 (PSGL-1), which binds to P-selectin on platelets, triggering intracellular signaling cascades that enhance Reactive Oxygen Species (ROS) production (51, 52). This interaction promotes Neutrophil Extracellular Trap (NET) formation, observed in acute myocardial infarction, which contributes to micro thrombosis and may worsen myocardial no-reflow after I/R injury (53, 54). Additionally, platelet—neutrophil binding activates integrins, such as Lymphocyte Function Associated Antigen 1 (LFA-1) and Macrophage -1- Antigen (Mac-1), which mediate adhesion to endothelial molecules like the Intracellular Adhesion Molecule 2 (ICAM-2), promoting neutrophil extravasation into the AAR (55). Platelets act as “binding bridges,” decelerating neutrophils and facilitating their transmigration into affected tissues via direct and indirect interactions with fibrinogen, GPIbα, and P-selectin (21, 55–57). Activated platelets interact with monocytes via P-selectin and glycoprotein Ib platelet subunit alpha (GPIb), triggering monocyte activation, degranulation, and transmigration into the AAR (18, 58–60). This also activates platelets to release Platelet Factor 4 (PF4) and Regulated And Normal T cell Expressed an Secreted (RANTES), enhancing monocyte adhesion and prolonging their lifespan, while PF4 stimulates macrophage inflammatory protein 1 alpha (MIP-1α), promoting monocyte differentiation into proinflammatory Ly6C^high^ macrophages (61–64).

In our data, PMCs were observed in both, the AAR and blood, Figures 4C,F. While no statistically significant changes were detected overall, anti-inflammatory Ly6C^low^ PMCs show a significant percentage decrease in the AAR during the first 7 days after I/R injury, Figure 4D. This reduction may reflect increased adherence of these complexes to the vascular endothelium, facilitating monocyte migration into the AAR rather than accumulation within the tissue or circulation.

Taken together, these findings suggest that the impact of PNCs and PMCs on myocardial regeneration and scarring may arise not from the complexes themselves but from their role in facilitating leukocyte infiltration and differentiation. It is conceivable that these complexes represent a transient intermediate state, reflecting a dynamic step in the immune cascade. Given the highly time-dependent nature of post-I/R immune responses, the detection of such complexes at specific time points may capture only a narrow but biologically meaningful window. Understanding these interactions may offer promising avenues for modulating the inflammatory response and mitigating reperfusion injury.

### 4.4 Echocardiographic assessment reflects observed immune cell changes after I/R injury

To place the cellular data in a clinically meaningful context and to confirm the technical success of the I/R procedure, echocardiography was performed at all time points studied. This allowed parallel assessment of myocardial kinetics, mobility and function. At the time of neutrophil and monocyte invasion into the area at risk (AAR) (Figures 2A,E) and during the dominance of pro-inflammatory subtypes within the myocardial tissue (Figure 3A), myocardial function, as represented by EF and FS, was significantly impaired compared to baseline from day 1 to day 7 post I/R injury (Figures 5A,B). As expected, this decline is consistent with previous reports (65). While these findings provide functional support for the observed temporal immune cell dynamics, a direct mechanistic link between cellular composition and myocardial function cannot be established at this time. Further studies are warranted to clarify the relationship between immune cell kinetics and functional cardiac recovery.

## 5 Limitations

The study's small sample size (five to six animals per group) limits statistical significance, though trends are apparent. Future research with larger sample sizes and additional time points—either extending beyond 7 days or focusing on the first 24 h could provide more definitive insights. Furthermore, we only ever have analyzed percentages of leucocytes.

## 6 Summary

### 6.1 I/R injury: immune dynamics and cardiac dysfunction

I/R injury triggers systemic immune activation, marked by neutrophil and monocyte infiltration into the AAR. Neutrophil proportions peak by day 3, exacerbating tissue damage via reactive oxygen species and protease release, while monocyte proportions, recruited through neutrophils, clear debris and transition to anti-inflammatory phenotypes to support repair. We could show that anti-inflammatory and regenerative functions are evident, not only in monocytes and macrophages, but also in neutrophils. Echocardiographic analysis shows impaired cardiac function, with reduced EF and FS within the first 24 h after I/R injury. During this periode we observed high levels of proinflammatory leukocytes and peak inflammation. PNCs and PMCs facilitate leukocyte migration and differentiation, indirectly influencing myocardial repair.

## 7 Conclusion

The phenotypic shifts in neutrophils and monocytes, driven by the bone marrow and spleen, are central to I/R injury outcomes. Modulating pro-inflammatory subsets offers therapeutic potential to limit cardiac damage and improve recovery.

## 8 Outlook

Future research should focus on the therapeutic modulation of immune cell phenotypes, particularly targeting specific neutrophil and monocyte subsets to mitigate tissue damage and promote regeneration. Furthermore, exploring the potential of platelet-leukocyte interactions as therapeutic targets could lead to novel strategies for enhancing myocardial repair and improving long-term cardiac function after I/R injury.

## Data Availability

The original contributions presented in the study are included in the article/Supplementary Material, further inquiries can be directed to the corresponding author.

## Supplementary material

Supplementary Figure S1 **CD206+ neutrophils differentiate in AAR following I/R**. Using the neutrophil-specific antibody CD206, activated antiinflammatory lineage+, CD11b+, Ly6Cintermediate, CD206+ neutrophil subpopulations were identified in the **(A)** AAR and **(B)** blood. The analysis was performed using flow cytometry. Pre-sorting of leukocyte population by using forward scatter (FSC) and side scatter (SSC) was performed. **(A,B)** display the geometric mean fluorescence intensity (GMFI) of CD206 on the Y-axis, with the time points of measurement represented on the X-axis.

Supplementary Figure S2 **Flow cytometric gating strategy- quantification of leukocytes in blood, spleen and bone marrow.** Single-cell suspensions from tissue and blood samples were analysed by flow cytometry. Initial gating was performed using side scatter area (SSC-A) versus forward scatter area (FSC-A) to identify viable cell populations based on size and granularity **(A)**. Subsequently, singlets were selected by gating FSC height (FSC-H) against FSC-A to exclude doublets and aggregates **(B)**. Leukocytes were identified by CD45 expression **(C)**. CD19^+^ B cells and CD3^+^ T cells were excluded from further analysis **(D)**. From the remaining CD45^+^ CD11b^+^ population, innate immune cells including monocytes, macrophages and neutrophils were selected **(E)**. Monocyte and neutrophil subtypes were distinguished based on CD115 and Ly6C expression **(F)**: CD115^+^ Ly6C^low^ and CD115^+^ Ly6C^high^ cells were classified as monocyte subsets, while CD115− Ly6C intermediate cells were defined as neutrophils. To further characterise neutrophil subtypes, the geometric mean fluorescence intensity (GMFI) of CD206 expression was measured within the neutrophil gate.

Supplementary Figure S3 **Flow cytometric gating strategy- quantification of PLCs in blood**. Leukocyte selection was performed (Supplementary Figure S2). Further subdivision of the leukocyte populations was based on Ly6G expression and cell granularity (SSC-A), allowing discrimination between Ly6G^+^ neutrophils, Ly6G- monocytes and lymphocytes **(A)**. To ensure a clear separation between monocytes and lymphocytes, an additional intermediate gating step was performed using SSC-H parameters. Subsequently, platelet-neutrophil-complexes (Ly6G^+^CD41^+^) and platelet-monocyte-complexes (PMCs; Ly6G-CD41^+^) were identified **(B–D)**.

Supplementary Figure S4 **Flow cytometric gating strategy- quantification of leukocytes and PLCs in heart tissue**. Flow cytometric analysis of leukocytes in the AAR was performed using a modified gating strategy to account for tissue-specific characteristics. Following initial selection of singlets **(A,B)**, CD11b^+^ monocytes and neutrophils were identified after exclusion of lineage-positive cells, including NK cells, B and T lymphocytes, and their precursors, as well as based on integrin expression **(C)**. Neutrophils were further subdivided into CD206^−^ pro-inflammatory and CD206^+^ anti-inflammatory subsets **(D)**. Platelet-neutrophil complexes (PNCs) were identified by co-expression of the platelet marker CD41 within these neutrophil subsets, allowing discrimination of CD206^−^ PNCs **(E)** and CD206^+^ PNCs **(E**′**)**. Macrophages were gated separately based on standard marker combinations **(F)**. Monocytes were further classified into Ly6C^high^ and Ly6C^low^ subtypes **(G)** and their association with platelets was assessed by CD41 co-expression to identify Ly6C^high^ PMCs and Ly6C^low^ PMCs **(H/H**′**)**.
