# Supplementary figures and images for: Shifting landscapes: dynamic changes from pro- to anti-inflammatory leukocyte phenotype in myocardial ischemia/reperfusion injury
Source: Front Cardiovasc Med. 2025 Jun 30;12:1596538. doi: 10.3389/fcvm.2025.1596538 (PMC12256526; doi:10.3389/fcvm.2025.1596538)

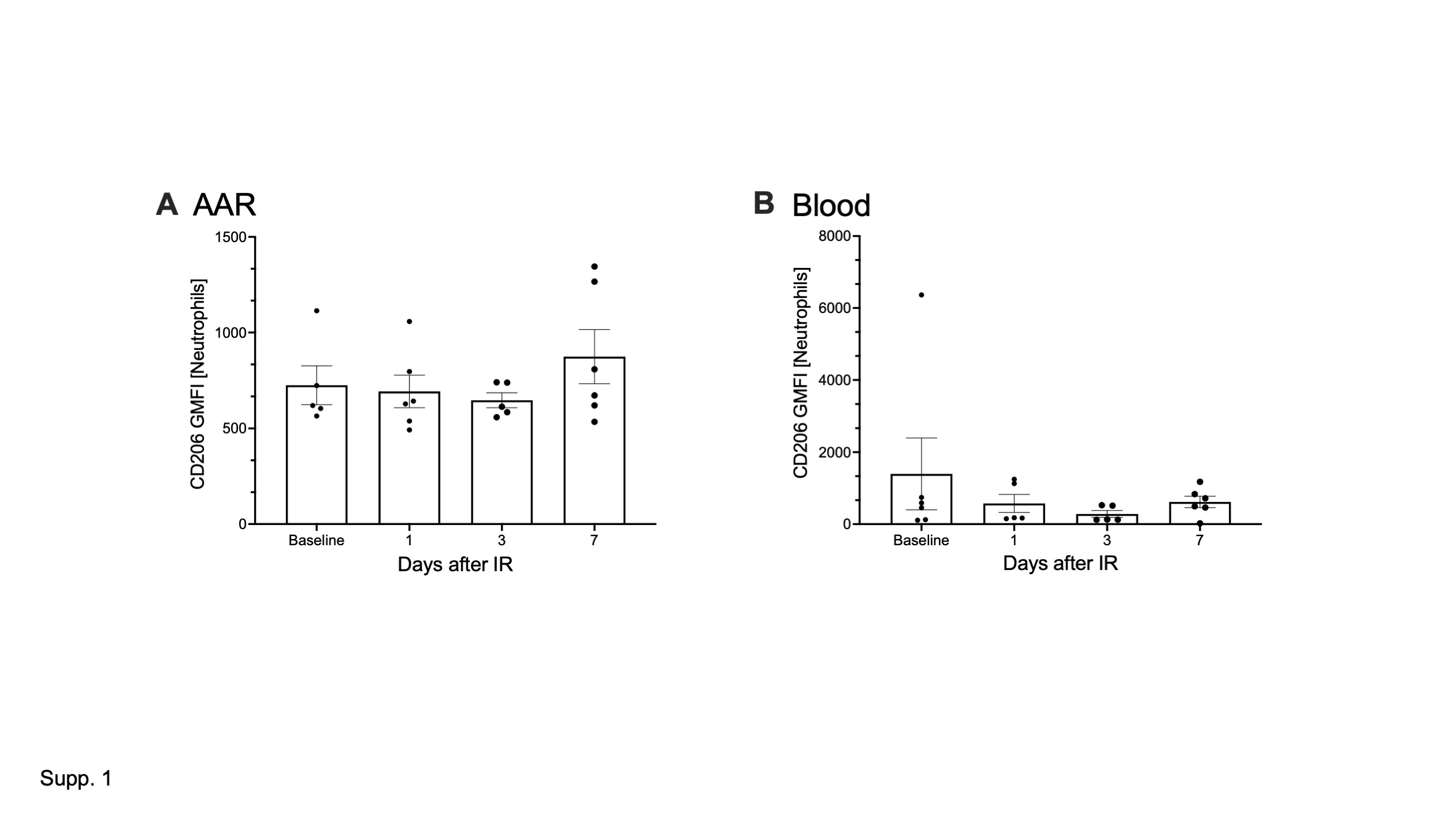

Supplement: Supplementary Figure S1 — CD206+ neutrophils differentiate in AAR following I/R. Using the neutrophil-specific antibody CD206, activated antiinflammatory lineage+, CD11b+, Ly6Cintermediate, CD206+ neutrophil subpopulations were identified in the (A) AAR and (B) blood. The analysis was performed using flow cytometry. Pre-sorting of leukocyte population by using forward scatter (FSC) and side scatter (SSC) was performed. (A,B) display the geometric mean fluorescence intensity (GMFI) of CD206 on the Y-axis, with the time points of measurement represented on the X-axis. [file Image1.jpeg]

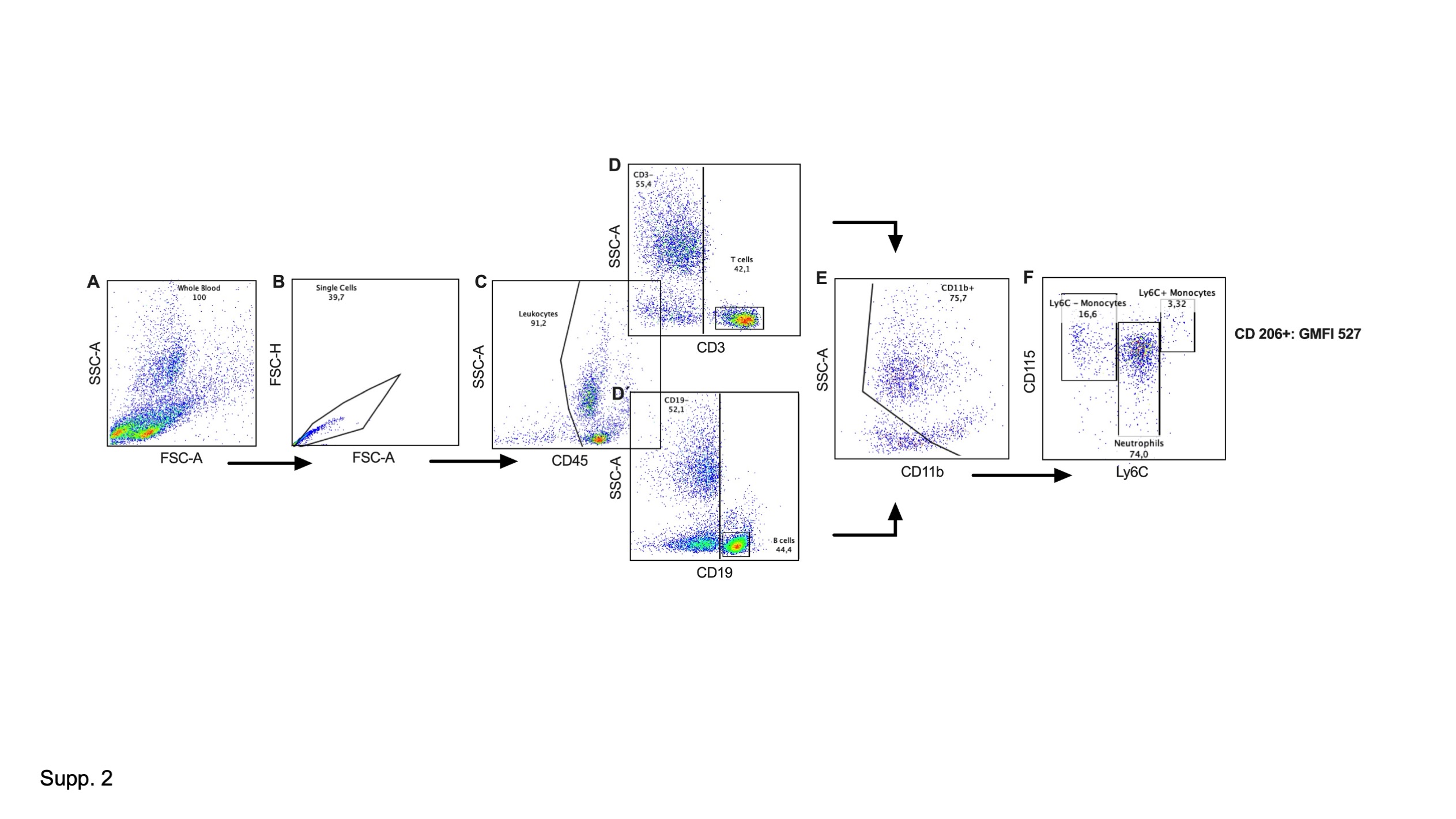

Supplement: Supplementary Figure S2 — Flow cytometric gating strategy- quantification of leukocytes in blood, spleen and bone marrow. Single-cell suspensions from tissue and blood samples were analysed by flow cytometry. Initial gating was performed using side scatter area (SSC-A) versus forward scatter area (FSC-A) to identify viable cell populations based on size and granularity (A). Subsequently, singlets were selected by gating FSC height (FSC-H) against FSC-A to exclude doublets and aggregates (B). Leukocytes were identified by CD45 expression (C). CD19+ B cells and CD3+ T cells were excluded from further analysis (D). From the remaining CD45+ CD11b+ population, innate immune cells including monocytes, macrophages and neutrophils were selected (E). Monocyte and neutrophil subtypes were distinguished based on CD115 and Ly6C expression (F): CD115+ Ly6Clow and CD115+ Ly6Chigh cells were classified as monocyte subsets, while CD115− Ly6C intermediate cells were defined as neutrophils. To further characterise neutrophil subtypes, the geometric mean fluorescence intensity (GMFI) of CD206 expression was measured within the neutrophil gate. [file Image2.jpeg]

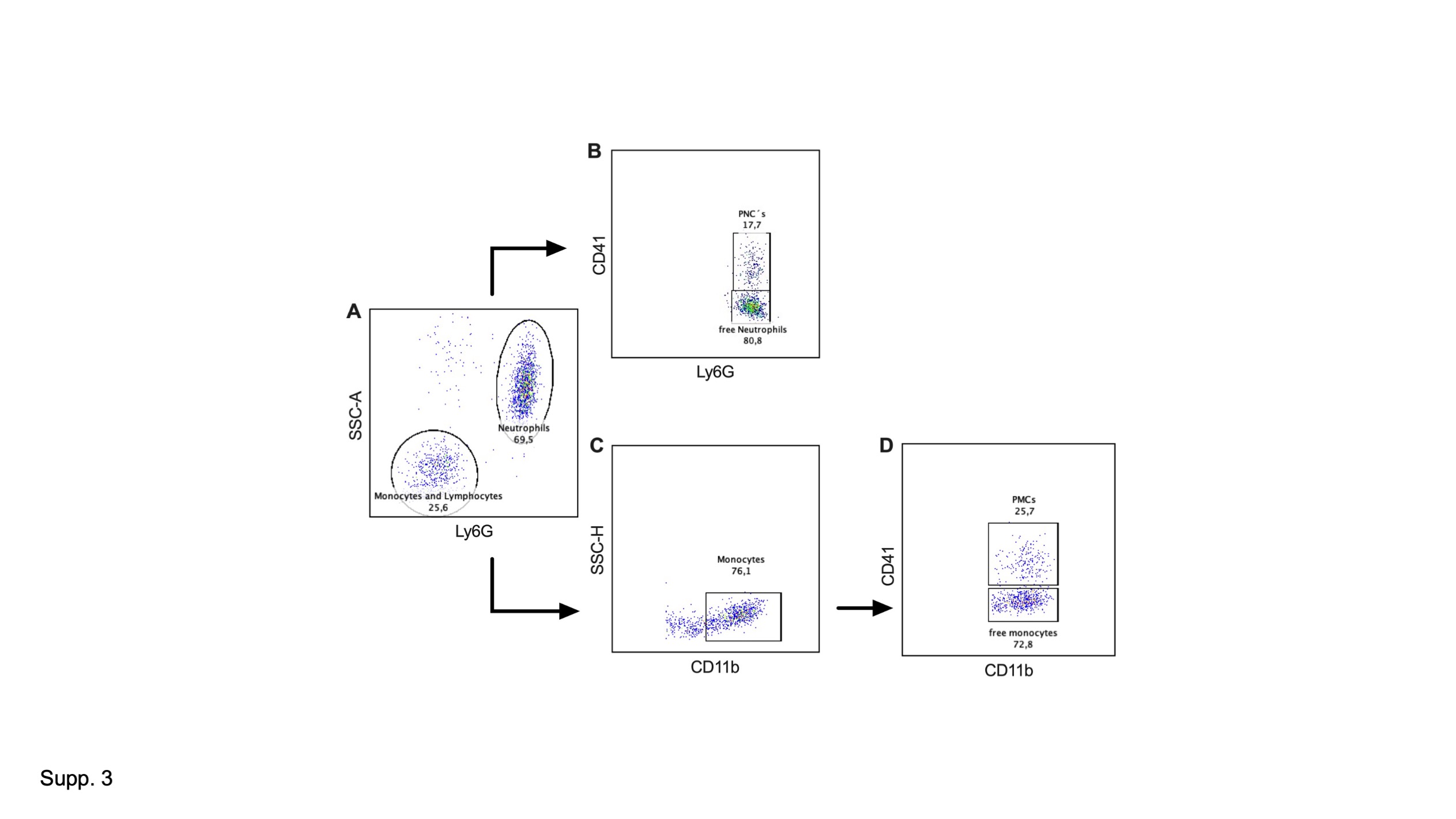

Supplement: Supplementary Figure S3 — Flow cytometric gating strategy- quantification of PLCs in blood. Leukocyte selection was performed (Supplementary Figure S2). Further subdivision of the leukocyte populations was based on Ly6G expression and cell granularity (SSC-A), allowing discrimination between Ly6G+ neutrophils, Ly6G- monocytes and lymphocytes (A). To ensure a clear separation between monocytes and lymphocytes, an additional intermediate gating step was performed using SSC-H parameters. Subsequently, platelet-neutrophil-complexes (Ly6G+CD41+) and platelet-monocyte-complexes (PMCs; Ly6G-CD41+) were identified (B–D). [file Image3.jpeg]

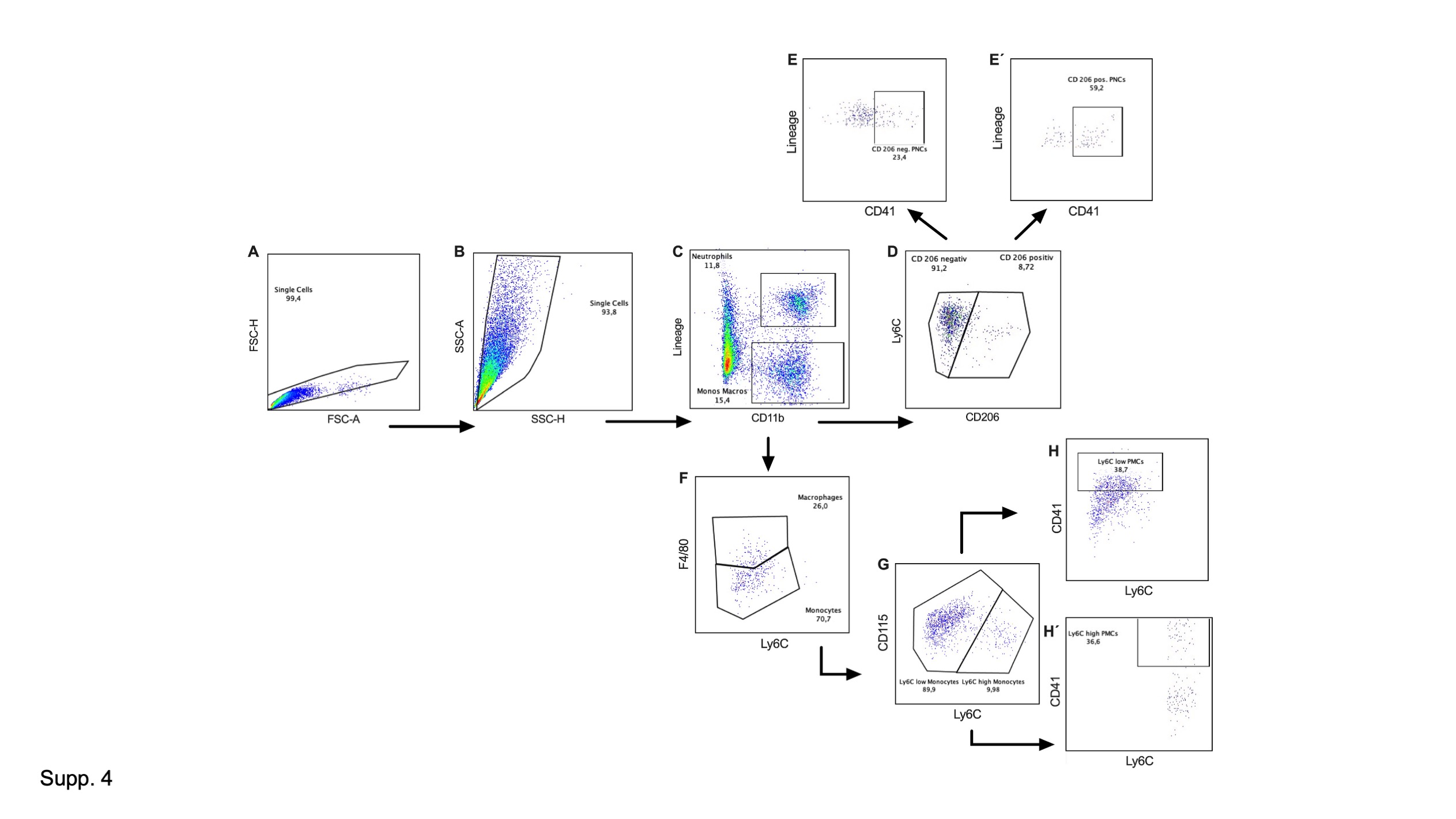

Supplement: Supplementary Figure S4 — Flow cytometric gating strategy- quantification of leukocytes and PLCs in heart tissue. Flow cytometric analysis of leukocytes in the AAR was performed using a modified gating strategy to account for tissue-specific characteristics. Following initial selection of singlets (A,B), CD11b+ monocytes and neutrophils were identified after exclusion of lineage-positive cells, including NK cells, B and T lymphocytes, and their precursors, as well as based on integrin expression (C). Neutrophils were further subdivided into CD206− pro-inflammatory and CD206+ anti-inflammatory subsets (D). Platelet-neutrophil complexes (PNCs) were identified by co-expression of the platelet marker CD41 within these neutrophil subsets, allowing discrimination of CD206− PNCs (E) and CD206+ PNCs (E′). Macrophages were gated separately based on standard marker combinations (F). Monocytes were further classified into Ly6Chigh and Ly6Clow subtypes (G) and their association with platelets was assessed by CD41 co-expression to identify Ly6Chigh PMCs and Ly6Clow PMCs (H/H′). [file Image4.jpeg]
